# New pattern of individualized management of chronic diseases: focusing on inflammatory bowel diseases and looking to the future

**DOI:** 10.3389/fmed.2023.1186143

**Published:** 2023-05-10

**Authors:** Xi Guo, Liyang Cai, Yuchen Cao, Zining Liu, Jiexin Zhang, Danni Liu, Zhujun Jiang, Yanxia Chen, Min Fu, Zhaoxia Xia, Guoguo Yi

**Affiliations:** ^1^Zhujiang Hospital, Southern Medical University, Guangzhou, Guangdong, China; ^2^School of Rehabilitation Sciences, Southern Medical University, Guangzhou, Guangdong, China; ^3^The Second Clinical School of Southern Medical University, Guangzhou, Guangdong, China; ^4^Plastic Surgery Hospital, Peking Union Medical College, Chinese Academy of Medical Sciences, Beijing, China; ^5^The First Clinical School of Southern Medical University, Guangzhou, Guangdong, China; ^6^The Third Clinical School of Southern Medical University, Guangzhou, Guangdong, China; ^7^The Second Clinical Medical College, Tianjin Medical University, Tianjin, China; ^8^The Sixth Affiliated Hospital of Sun Yat-Sen University, Guangzhou, Guangdong, China

**Keywords:** inflammatory bowel disease, chronic disease management, closed-loop prediction model, negative feedback, diagnostic prediction model

## Abstract

Non-infectious chronic diseases, especially inflammatory bowel diseases (IBDs), hypertension, and diabetes mellitus, are characterized by a prolonged and multisystemic course, and their incidence increases annually, usually causing serious economic burden and psychological stress for patients. Therefore, these diseases deserve scientific and consistent disease management. In addition, the lack of a comprehensive “early disease clues tracking—personalized treatment system—follow-up” model in hospitals also exacerbates this dilemma. Based on these facts, we propose an individualized prediction management system for IBDs based on chronic diseases, focusing on the established IBDs-related prediction models and summarizing their advantages and disadvantages. We call on researchers to pay attention to the integration of models with clinical practice and the continuous correction of models to achieve truly individualized medical treatment for chronic diseases, thus providing substantial value for the rapid diagnosis and adequate treatment of chronic diseases such as IBDs, which follow the “relapse-remission” disease model, and realizing long-term drug use and precise disease management for patients. The goal is to achieve a new level of chronic disease management by scientifically improving long-term medication, precise disease management, and individualized medical treatment, effectively prolonging the remission period and reducing morbidity and disability rates.

## 1. Introduction

Non-infectious chronic diseases, especially cancer, diabetes, inflammatory bowel diseases (IBDs), hypertension, coronary heart disease, and chronic obstructive pulmonary disease, require long-term treatment and meticulous care, usually causing a serious economic burden and psychological pressure on patients’ families (1, 2). If the treatment of these non-infectious chronic diseases is delayed or ineffective, worse disease progression can lead to more energy and financial resources being invested in the screening, diagnosis, and treatment of chronic diseases (3, 4).

With the development of medical technology and disease management systems, referring to the relatively complete process from preliminary diagnosis to clinical decision-making to personalized treatment (5), mortality caused by chronic diseases has decreased in high-income countries, and the speed is significantly faster than that for low- and middle-income countries (6). Simultaneously, there is evidence showing that, in low-income countries, the risk of death from non-infectious chronic diseases is twice that in high-income countries (7). The high mortality rate and low development speed undoubtedly aggravate the economic burden on low-income countries’ people. One reason is that, compared with areas with more developed chronic disease management, on the one hand, the environment in low-income areas is poor, and eating and living habits are poor; on the other hand, more importantly, screening for chronic diseases in these areas is not very common. The extent of diagnostic delay in low- and middle-income countries might be longer in comparison to high-prevalent regions in the West due to a lack of disease awareness (8). Moreover, the lack of nursing technology and medical conditions are also key factors rendering chronic disease management unsatisfactory. Therefore, a non-invasive, convenient, and effective management model for the whole process of chronic diseases that does not depend on high-end equipment is necessary, especially for low-income areas and populations.

Inflammatory bowel diseases, which include two main subtypes, namely Crohn’s disease (CD) and ulcerative colitis (UC), are always defined as a chronic inflammatory disease of the intestine caused by a complex interplay of genetic factors, dysfunctional host immune responses, and environmental triggers (9). Because of the complexity of the pathogenesis of IBDs, their diagnosis has always been an important challenge in clinical practice. Therefore, the diagnosis and prediction of IBDs have always been the focuses of clinical exploration. Accumulated clinical studies focused on the exploration of diagnosis and prognosis prediction of IBDs, for example, from biochemical (10) or immune (11) indicators screening the independent predictors and building the prediction models. The incidence of IBDs in Western countries is increasing annually (8, 12). With the recent development of globalization, the incidence of IBDs in Asian countries has also been increasing annually due to factors such as westernization of diets, greater industrialization, and increased pressure of life rhythms. To sum up, the occurrence, development and relapse of IBDs not only bring economic burden to the society, but also the patient’s psychological state is considered to be greatly affected (13). In addition, the individualized difference of patients’ response to treatments (14) also suggests that we need personalized diagnosis and treatment plan for IBDs. Therefore, a disease management model of IBDs, a chronic digestive system disease that places huge economic and psychological burdens on patients, is urgently needed.

**Figure 1:**
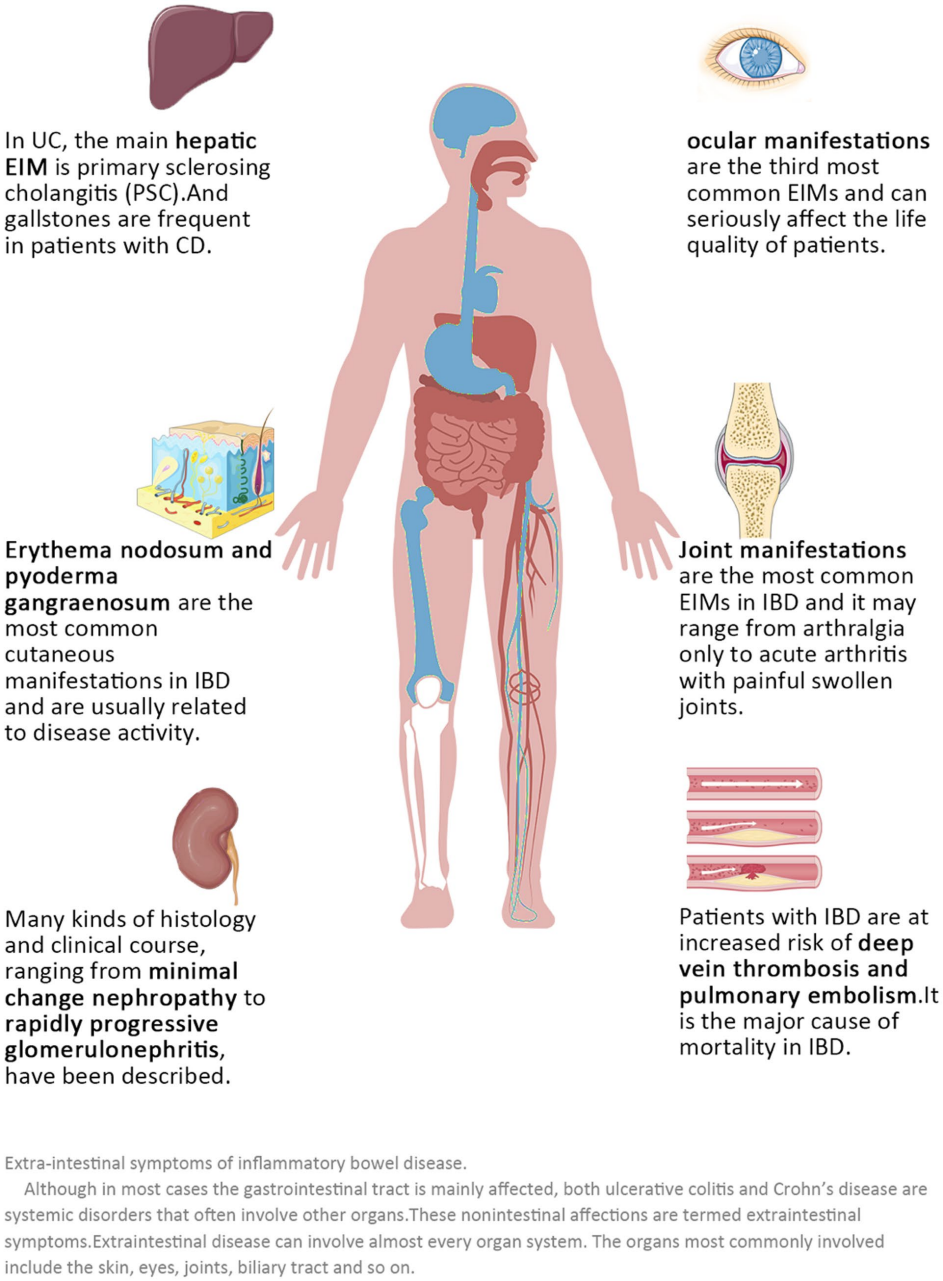
Extraintestinal symptoms of inflammatory bowel diseases. Although in most cases the gastrointestinal tract is mainly affected, both ulcerative colitis and Crohn’s disease are systemic disorders that often involve other organs. These non-intestinal effects are termed extraintestinal symptoms. Extraintestinal disease can involve almost every organ system. The organs most commonly involved include the skin, eyes, joints, and biliary tract, among others.

For patients with active IBDs, gastrointestinal manifestations should be the basis for disease assessment and diagnosis of IBDs (15). However, since IBDs are systemic diseases with a “relapsing–remitting” model, we should not only focus on the gastrointestinal reactions during the active phase but also note that many patients have many extraintestinal manifestations (EIMs) during the remission phase (16, 17). Given that EIMs are often associated with reduced quality of life in affected patients and predict mortality and relapse (18–21), rapid diagnosis and adequate treatment are needed. Overall, 6–47% of patients with IBDs have one or more EIMs (Figure 1), and almost all organs can be involved, posing a significant challenge to physicians managing patients with IBD (18, 22–25). If an individualized predictive management system for extraintestinal symptoms of IBDs patients can be established, both doctors and IBDs patients could pay sufficient attention to possible extraintestinal diseases, including IBDs eye diseases, and help to improve the quality of life of IBDs patients.

Based on chronic diseases, focusing on IBDs, we evaluate the established IBDs-related prediction models, summarize their advantages and disadvantages, and integrate all of the prediction models to propose the establishment of an individualized prediction management system for IBDs. We call on researchers to pay attention to the combination of model and clinical practice and to the continuous correction of the model and to strive to achieve true personalized medicine for chronic diseases.

## 2. Current condition of predictive model studies for IBDs

Patients with IBDs, especially those with CD, are prone to underdiagnosis and misdiagnosis due to their atypical clinical presentations (12). Therefore, the screening of reliable predictors and the establishment of accurate clinical prediction models (CPMs) are of great importance in guiding clinical diagnosis and treatment (26). The epidemiological features also suggest that a multifaceted and individualized approach to prevention, treatment, and reduction of recurrence, combined with relevant influencing factors leading to comprehensive precision medicine and personalized treatment, is essential for the management of this complex disease, which has a serious impact on the quality of life of patients. However, although this treatment model has been expected in the field of IBDs treatment, due to the complexity of the etiology and pathogenesis, IBDs have been considered a “difficult to diagnose, difficult to predict” diseases, and mature predictive models have rarely been reported.

### 2.1. Diagnostic model in IBDs

#### 2.1.1. Diagnostic model by immune component factors

A major challenge in IBDs is currently the integration of different IBDs data sets to construct predictive models of IBDs. Unlike previous genetic-level studies, Peters et al. proposed a predictive model for the immune component of IBDs that uses functional and regulatory annotations associated with the cellular, histological, and pathophysiological aspects of IBDs to inform causal relationships between motifs previously associated with IBDs through genome-wide association studies (GWASs) (27). This validated set of key drivers not only introduces new regulators for the core processes of IBDs, but it also provides directly interrogable ICs for genetic, molecular and clinical characteristics to determine and refine the regulatory framework that defines IBDs.

However, this model was only validated in isolated cells and mice, it is undetermined how these differences compare in homeostasis and inflammatory disease. Moreover, immune component factors are highly interrelated, and the expression of many factors can be altered by perturbations of others. It is the more important refinements that need to be made as the model is evolved.

#### 2.1.2. Diagnostic model by multiomics of stool samples

The disadvantage of invasive tests is that patients must bear more pain, risk, and cost. However, for the diagnosis of IBDs, noninvasive testing methods must still be developed and refined (28). Noninvasive testing and artificial intelligence modeling analysis using stool and blood samples hold the promise of resolving the diagnostic dilemma for patients with IBDs. Franzosa et al. developed a random forest model based on multiomics data from stool samples for the recognition of CD, UC, and non-IBDs (29). However, there is still room for improvement in the three existing staging diagnostic models of non-IBDs, CD, and UC. Therefore, Huang et al. developed an artificial intelligence diagnostic model based on six-dimensional multiomics data using stool specimens from non-IBDs, CD, and UC groups as samples to provide more noninvasive diagnostic techniques for patients with IBDs with different self-evaluation statuses (30). This method allows for the diagnosis and staging of IBDs by simply collecting a stool specimen. It is highly accurate, is noninvasive, and does not require cumbersome and painful clinical colonoscopy and biopsy.

Relevant diagnosis and treatment guidelines indicated that fecal biomarkers such as fecal calprotectin and fecal lactoferrin can be used as biomarkers for differential diagnosis of IBD (31), and can be used as markers for UC monitoring and management (31). Fecal biomarkers are also suggested as good indicators for excluding gastrointestinal infections (15). The recommendations of the above guidelines provided us the confidence that stool monitoring as a non-invasive examination method has good diagnostic and disease monitoring potential.

#### 2.1.3. Diagnostic model by serum proteomics

With the rapid development and close integration of molecular biology, computers, and information technology, human research into diseases has entered the era of molecular hematology, making possible the development of biological markers for serum proteomics. Serum protein fingerprinting is a novel proteomics technique developed in recent years to search for disease-related proteins, to study the protein expression or posttranslational modifications that lead to diseases as a whole, to find diagnostic markers for IBDs, to establish a diagnostic model for IBDs in peripheral blood, to reflect on the occurrence and development of IBD, and to open new pathways for early clinical diagnosis and prognosis of IBDs.

Zhang et al. (33) used weakly cationic magnetic beads combined with MALDI-TOF-MS to analyze the serum proteomic profiles of IBDs patients and normal controls and found that differentially expressed protein peaks were effective in distinguishing the IBDs group from normal controls and the CD group from the UC group. At the same time, the genetic algorithm was combined with the SVM to build the diagnostic models of serum differential protein peaks for the CD group and normal control group (four combinations of protein peaks with M/Z of 4,964, 2,272, 2,126, and 2,900), serum differential protein peaks for the UC group and normal control group (six combinations of protein peaks with M/Z of 3,030, 5,065, 2,360, 3,275, 1,945, and 3,957), and the specific diagnostic models of serum differential protein peaks for the CD group and UC group (four combinations of protein peaks with M/Z of 2,900, 5,338, 2,122, and 1,897). The specificity and sensitivity of the serum differential protein peak diagnostic model (M/Z of 2,900, 5,338, 2,122, and 1,897) between the CD group and the UC group were high, which could assist in the early diagnosis of IBDs and the differential diagnosis between its subtypes (32). This result, however, is subject to further experimentation using a larger sample cohort.

In addition, a preclinical diagnostic prediction model for IBDs have been developed to predict patients who will be diagnosed with CD within 5 years with high accuracy by testing a panel of serum antibodies and proteins in patients, but the predictive efficacy of the relevant serum antibodies and proteins used to diagnose UC is not satisfactory (10).

#### 2.1.4. Diagnostic model by machine learning

Poor accuracy, inefficiency, and loss of early screening have prompted the development of new UC prediction models. With advances in technology, machine learning has emerged as a new approach to medical data processing, such as random forest (RF) and artificial neural networks (ANNs). For RF, it builds Bagging integration based on a decision tree (DT) and further introduces random attribute selection in the training process of DT. This method can effectively improve the classification accuracy of new samples. RF algorithms are well-established in the field of Alzheimer’s disease and acute myeloid leukemia (33, 34). ANNs are the computational model networks that broadly simulate the functions of the human cerebral cortex and can replicate similar ways of thinking and perception to humans. A neural network is a hierarchical structure composed of interconnected nodes that contain activation functions for calculating the output of the network (35). ANNs have also shown powerful capabilities in medical data processing (36).

Thus, Li et al. used RF technology to discover UC susceptibility genes, built an integrated toolkit of genes associated with UC disease occurrence, and developed a combination of machine learning algorithms to introduce a new clinical predictive score, the MPS, for general UC patients, resulting in an ANN predictive model that can provide early screening indicators for the diagnosis and treatment of UC (37).

Innovative combinations of machine learning methods can be used to improve the predictive power of UC prediction models and achieve better creative prediction results. As an integrated algorithm, RF has shown excellent performance in handling highly accurate and precise multifeature data. RF algorithms have been widely used for the detection and prediction of clinical diseases. However, in this study, the MPS system and the prediction model was based on the “Gene Expression Omnibus” (GEO) data set only; therefore, its prediction model should be further evaluated and validated in laboratory experiments and clinical work. It is also noted that the development of this new prediction model did not exclude the influence of environmental factors on UC, so its new prediction model based on susceptibility genes might to some extent have the same limited predictive power as other prediction models.

### 2.2. Prognostic model In IBDs

Prognostic models are models that predict the outcome of related diseases and are commonly used to predict the progression status of a disease, patient survival time, and the probability of developing to a disease stage in clinical practice. The predictors and prediction methods are diverse, including radiomics, autophagy-related genes, DNA methylation, noncoding RNA, alternative splicing, and protein disulfide isomerase (PDI). The above factors construct IBDs prognostic models in a single or combined manner, and clinical characteristics are often combined with other factors.

#### 2.2.1. Prognostic model by mathematical model

A mathematical model (MM) is a tool that imitates reality by using the language of function. It is often represented as a scoring system in medicine. The efficiency of MM is directly reflected in its specificity and sensitivity. Based on the importance of imaging techniques in the process of diagnosing IBDs, some scholars have also constructed relevant MM at the imaging level can assist in the diagnosis, evaluation, and prognosis.

The Crohn’s disease endoscopic index of severity (CDEIS) is a scoring system for endoscopists to assess the severity of CD lesions, using the GELS score as the dependent variable and the lesion observed by endoscopy as the independent variable (38). The CDEIS has been validated and reproduced with multicenter data and can consistently predict the severity of CD lesions (38). However, the scoring model is relatively complex and time-consuming and lacks a valid threshold for the classification of severity (39).

The simple endoscopic score for Crohn’s disease (SES-CD) is a simpler scoring model than the CDEIS for endoscopic evaluation of the severity of CD (39). A multiple linear regression approach was used to model the CDEIS score as the dependent variable and the scores for ulcer size, ulcer surface, affected surface, and canal stenosis (0–3) as the independent variables. It is easier for endoscopists to assess CD severity using the SES-CD scoring model than the CDEIS, and the SES-CD correlates well with the CDEIS and fecal calprotectin (39, 40). It is also more suitable for everyday clinical work.

Paddington International Virtual ChromoendoScopy ScOre (PICaSSO) is the first model to evaluate the characteristics of the healed blood vessels and mucous membranes using electronic virtual chromocopy (VEC). PICaSSO score showed good consistency, and was considered can be used to define the endoscopic results of UC mucosa and vascular healing, and reflect the full range of histological changes (41). In the following real world multi-center studies, there was a strong correlation between PICaSSO and histological scores, which could predict specific clinical outcomes at 6 and 12 months. Therefore, PICaSSO can be a useful endoscopic tool in UC treatment management. Moreover, PICaSSO performs better than traditional endoscopy scores (42). However, as this is a newly developed scoring method, there are relatively few studies on it and most of them are limited to experienced endoscopic physicians. Further evaluation is needed for this promising scoring in the future.

The magnetic resonance index of activity (MaRIA) is a model for the quantitative assessment of CD disease activity using MRI (43). MaRIA uses MRI to assess CD activity; its scores are relatively objective and reliable, and the validity of MaRIA has been demonstrated (44). Using methods such as receiver operating characteristic (ROC) curves and bootstrap analysis to analyze the model, which also determines thresholds, sensitivity, specificity, and parameter accuracy (44, 45), MaRIA is also effective in assessing the responses of CD patients to treatment (46). The Clermont score is an assessment model that uses the DWI sequence in MRI to assess the severity of CD (47). The advantage over MaRIA is that it does not require injection of gadolinium contrast, but its clinical value requires further confirmation (47).

Alam et al. developed a simple diagnostic differential model by screening for 18F-FDG PET/CT signs that could be used to differentially diagnose PIL from CD (48). However, the small sample size of included cases due to the rare clinical nature of PIL makes it difficult to exclude the existence of study bias. To further investigate the generalizability of the model and to explore other signs that could have potential value, the sample size should be further increased and justified by a multicenter study.

However, these models are dependent on clinical symptoms, endoscopy, and imaging findings, which are also limited to subjective experience of clinicians. There are also defects such as difficulty in obtaining indicators or high expense.

Other mathematical models, such as the Crohn’s disease activity index (CDAI) and the simplified Crohn’s disease activity index (including the HBI and short CDAI), are also used to assess the activity, severity, and lesions at the time of IBDs. The Rutgeerts score is the only model that has been used to predict recurrence in patients with CD after surgery, and it still has major limitations in predicting recurrence in IBDs, mainly because it can answer the qualitative questions only as yes or no but not quantitatively for certain clinical outcomes (49). Moreover, there is a lack of models for diagnosing and predicting the prognosis of IBDs in the clinical setting. This requires more research by clinicians and medical researchers to develop new mathematical models.

#### 2.2.2. New breakthroughs in IBDs prognostic models

Fecal biochemical markers, protein biomarkers (e.g., calprotectin), and metabolomic markers offer a greater choice of variables for the prediction of IBDs recurrence, but they also have major limitations, and there is no established model for predicting recurrence. In the Republic of Korea, the Korean Crohn’s disease prediction (KCDP) model was developed using training set data and validated with a validation set (50). This model is the first one validated for predicting surgical risk in South Korean CD patients; it provides an accurate individualized estimate of the probability of surgery using clinical parameters collected at diagnosis, which can guide early intensive treatment of CD and the selection of appropriate patients. However, due to the great geographical variability in the onset of IBDs, further validation of this model for extrapolation to other regions is needed.

Scientific and technological advances in the field of research have led to the systematization of several sets of molecular prognostic indicators associated with UC. The mRNA for neutrophil gelatinase-associated lipid transport protein (NGAL) is overexpressed in the inflamed intestine. Therefore, Budzynska et al. used NGAL to predict clinical and endoscopic activity in UC (area under the curve [AUC] = 0.758) (51). Hart et al. combined fecal calprotectin (FC) with the Mayo Endoscopy Score (MES) to predict endoscopic and histological activity in UC patients in clinical remission (AUC = 0.743) (52). However, these predictive models are not sufficiently effective in the screening and early diagnosis of UC, considering that clinical outcome assessment is based on a range of laboratory tests and procedures. A valid and general predictive model for the early identification and intervention of UC is lacking.

In summary, although IBDs are still considered “difficult to diagnose, difficult to predict” diseases due to the complexity of the etiology and pathogenesis, various prediction models have emerged. Whether from a technological breakthrough; by choosing different levels of research, such as susceptibility genes or immunological perspectives; or by building mathematical models with the help of mathematical modeling methods that focus on different stages of disease development and characterization, these models have, to some extent, contributed to the early identification, diagnosis and prognosis of IBDs and are of great importance for the differential diagnosis, outcome and regression prediction of IBDs (Tables 1, 2).

**Table 1:** Application of diagnostic model in IBDs distinguish.

Method	Name	Year	Model	Innovations in model design	Meaning
Immune ingredient	Lauren A. Peters	2017	CIC IBDs network	Integrate large-scale DNA and RNA variation data in the context of active IBDs to construct a model of the pathological inflammatory component of IBDs	The first demonstration of a model constructed from IBDs intestinal tissue sourced from three distinct patient populations
Multiomics of stool samples	Eric A. Franzosa	2019	Random forest model based on multiomics data from fecal samples	Multiomics data from stool samples	For diagnosis of CD, UC, and non-IBDs
	Qiongrong Huang	2021	Artificial intelligence model based on fecal multi-omics data	Stool specimens from the non-IBDs, CD, and UC groups	Provided a valuable method for high accuracy, noninvasive diagnosis and subtype identification of IBDs patients
Serum proteomics	Fenming Zhang	2016	Diagnostic models of serum differential protein peaks	Used weakly cationic magnetic beads combined with MALDI-TOF-MS to analyze the serum proteomic profiles of IBDs patients and normal controls	Assisted in the early diagnosis of IBDs and the differential diagnosis between its subtypes
	Joana Torres	2020	Multivariate model based on serum biomarkers	Used biomarkers to identify patients at risk for development of inflammatory bowel diseases	Identify patients who will develop CD up to 5 years before diagnosis
Machine learning	Hanyang Li	2020	Artificial neural network predictive model	Used RF technology to discover UC susceptibility genes, built an integrated toolkit of genes associated with UC disease occurrence, and developed a combination of machine learning algorithms to introduce a new clinical predictive score, the MPS, for general UC patients	Provided early screening indicators for the diagnosis and treatment of UC

**Table 2:** Application of prognostic model in IBDs distinguish.

Method	Name	Year	Meaning	Shortage
Prognostic model by mathematical model				
CDAI	NCCDS	1976	The gold standard for CD activity	More items, longer duration of symptoms (1 week), more dependent on the subjective judgment of the physician
HBI	R. F. Harvey	1980	Fast evaluation and easy to popularize	Efficacy and specificity have not been demonstrated by prospective studies
Rutgeerts score	Rutgeerts P.	1984	The only model that has been used to predict recurrence in patients with CD after surgery	Answer the qualitative questions only as yes or no but not quantitatively for certain clinical outcomes
MES	Schroeder KW	1987	It scores the severity of UC endoscopic lesions based on endoscopic findings. The simple and easy operation of MES has made it widely used in clinical practice	MES only considers the severity of endoscopic lesions, not the extent of the lesions
CDEIS	J. Y. Mary	1989	Predicted the severity of CD lesions consistently	Complex, time-consuming and lacks a valid threshold for the classification of severity
SES-CD	Marco Daperno	2004	A simple, reproducible, and easy-to-use endoscopic scoring system for Crohn’s disease	/
MaRIA	J. Rimola	2009	Using MR as an alternative to endoscopy in the evaluation of ileocolonic Crohn’s disease	In patients with resected segments an underestimation of the global score occurs
Short CDAI	Kelvin Thia	2010	Requires far fewer items than CDAI, facilitating clinical application	It still takes 1 week and relies heavily on subjective evaluations
UCEIS	Simon Travis	2012	Highly correlated with the severity of the overall assessment. The use of UCEIS is simple and can reliably assess the overall endoscopic severity of UC	/
Clermont score	C. Hordonneau	2014	Not require injection of gadolinium contrast	Clinical value require further confirmation
New breakthroughs in IBD prognostic models				
MMES	Lobatón T.	2015	Breaking the limitation that MES does not consider the degree of inflammation and therefore cannot achieve segmental endoscopic improvement	/
KCDP model	Yehyun Park	2017	To guide early intensive treatment of CD and selection of appropriate patients	Need further validation for extrapolation to other regions
NGAL	Budzynska	2017	To predict clinical and endoscopic activity in UC	Not sufficiently effective in the screening and early diagnosis of UC
Combined FC with the MES	Hart	2020	To predict endoscopic and histological activity in UC patients in clinical remission	
Artificial intelligence computer-aided system	Marietta Iacucci	2023	The artificial intelligence model can help distinguishes histological remission/activity in biopsies of UC and predicts flare-ups. This can expedite, standardize and enhance histological assessment in practice and trials.	/

## 3. Current status of predictive models for extraintestinal symptoms of IBDs

From the above, we know that IBDs should be considered a systemic disease not limited to the gastrointestinal tract since many patients will present with extraintestinal symptoms. Extraintestinal symptoms can involve virtually any organ system and have a potentially detrimental effect on the functional status and quality of life of the patient. Extraintestinal symptoms can be divided into two groups: extraintestinal manifestations (EIMs) and extraintestinal complications. Early recognition of EIMs is important because they can characterize subclinical inflammation in patients with IBDs and increase the probability of mortality and recurrence. Extraintestinal complications are mainly caused by the disease itself and include malabsorption and subsequent micronutrient deficiencies, osteoporosis, IBDs drug-related side effects, and other conditions. Due to extraintestinal complications’ broad scope and harmful effects on patients, the study of related prediction models has progressed slightly ahead of EIMs.

### 3.1. Predictive models for EIM

Because EIMs has a negative impact on patient prognosis, predicting possible risks by identifying independent and relevant predictors is the first step to ensuring long-term patient health (53), and understanding the risk of EIMs associated with patient characteristics is important for better planning and more tailored treatment strategies (54). However, predicting the occurrence of EIMs based on patient characteristics is a challenging task for two main reasons: the increasing amount of information about subject characteristics and the limited number of patients for whom valid data are available (55).

#### 3.1.1. Traditional statistical models

It is well known that the most classical and commonly used statistical tools, such as linear regression models, have limitations in multivariate situations. In addition, some risk factors are known to have a nonlinear effect on the likelihood of EIMs, and they interact with other covariates, such as IBD1 vs. IBD5 (56) and family history (57). These facts can all limit the use of traditional statistical models in predicting EIMs. Giachino et al. selected several traditional statistical models using Akaike information criteria (logistic regression as a benchmark, generalized additive model, and projection pursuit regression, linear discriminant analysis, and quadratic discriminant analysis) and compared the accuracy of the various models by means of an array of metric systems (58). The results showed that the overall predictive power of these models was impressive, especially after accounting for genetic factors, with approximately 90% of the AUC being quite similar to all of the models considered; among them, projection pursuit regression (PPR) performed better than the other models, which can be attributed to the nonlinear effect of the principal component transformation of the covariates by PPR for the inherent ability of modeling, which is of particular interest in the context of unsupervised data mining (59).

Therefore, we can assume that the current traditional model still has strong application value. In particular, the performance of PPR is impressive and has the potential to be further explored and gradually applied in clinical practice.

#### 3.1.2. Machine learning-based predictive models

The high complexity of EIM prediction requires more robust and sophisticated statistical methods that provide valuable information from a clinical perspective. Indeed, traditional statistical methods might not represent the best solution for characterizing this complex risk structure, which can often be characterized by nonlinear relationships between outcome and predictor variables and interactions between covariates. Instead, traditional machine learning techniques (MLTs) have been promoted as a promising approach for modeling EIM predictions, including genetic factors (58). Recently, integrated Bayesian frameworks have been proposed in the MLT domain, and these methods are able to combine the modeling flexibility of MLTs with the benefits of Bayesian inference, making them potentially superior in situations in which standard methods might fail, such as when addressing missing data, small sample size data, and embedded external information (60).

Menti et al. evaluated the predictive performance of three different techniques (plain Bayesian, Bayesian additive regression trees, and Bayesian networks) as classifiers for the extraintestinal manifestations of 152 CD patients; the results showed that Bayesian networks achieved 82% accuracy when only clinical factors were considered and 89% when genetic information was considered, outperforming the other techniques but with little predictive power for CD14 (61). However, when Bottigliengo et al. (62) evaluated all three techniques equally, they found that BMLT performed worse than expected in classifying the presence of EIMs when mixed genetic and clinical data were available, but relevant data were also missing compared to classical statistical tools, as often occurs in clinical practice. Moreover, the study by Giachino et al. also showed that single-layer ANNs did not show any particular improvement in model accuracy compared to nonlinear techniques.

In summary, we believe that machine learning-based prediction models have good application prospects, but the current prediction results remain poor, and better code design and larger amounts of clinical data might be able to improve their performance.

### 3.2. Predictive model for extraintestinal complications

#### 3.2.1. Use in acute care stratification management

Most patients with acute CD will undergo abdominal CT in the emergency department (ED) to exclude disease complications. Khoury et al. developed a simple noninvasive scoring model to predict the presence of intra-abdominal abscesses in patients with acute CD. This score can be used as an aid in the emergency stratification of patients with acute CD, in whom the likelihood of the presence of intra-abdominal abscesses is high (63). In addition, studies have shown that multiple risk factors during disease progression can lead to a prothrombotic state (PTS), which predisposes patients to thrombosis. Therefore, predicting PTS can help to identify patients at risk for thrombosis. Pan et al. classified CD patients by D-dimer levels and constructed a predictive model for PTS with high accuracy for the diagnosis of PTS in CD patients (64).

#### 3.2.2. Predicting the risk of cancer

In addition, IBDs can be combined with colorectal cancer (CRC). Therefore, Andersen et al. started from the current state of research on the oncogenic mechanisms of IBDs and the genetic susceptibility of IBDs patients in an attempt to combine genotypic and clinical parameters into a predictive model that would refine the prediction of CRC risk in colonic IBDs (65). Extensive genetic alterations are present not only in the tumor lesions associated with UC but also in the adjacent normal colonic mucosa. This fact suggests that genetic changes in the nonneoplastic mucosa might be useful markers for predicting the occurrence of UC-associated cancer (UC-Ca). Watanabe et al. developed a predictive model for the occurrence of UC-Ca based on gene expression levels in the nonneoplastic rectal mucosa measured by reverse transcription polymerase chain reaction (RT–PCR) analysis (66). This predictive model suggests that patients with UC are at high risk for developing cancer. These results have important implications for improving the effectiveness of colonoscopy and point to future research on the molecular mechanisms of UC-related cancers (Table 3).

**Table 3:** Representative application of predictive models in extraintestinal symptoms of IBDs.

Name	Year	Related technology	Innovations	Meaning
Daniela F. Giachino	2007	Inefficient statistical models in genetic factors	Statistical and comparative predictive performance of six of the most classic statistical tools	①PPR performs better than other models;
				②Single-layer artificial neural networks do not show any improvement in model accuracy compared to nonlinear techniques
Toshiaki Watanabe	2011	Genetic analysis	Predicting the risk of colorectal cancer from IBD in nonneoplastic rectal mucosa	Helps to improve the effectiveness of colonoscopy and provides a direction for future research on molecular mechanisms
E. Menti	2017	Machine learning techniques	The predictive performance of three techniques was evaluated in comparison with traditional statistical tools	①Bayesian analysis is a viable and accurate tool for predicting EIMs
				②IL and TFN genes increase prediction accuracy by 10%
Daniele Bottigliengo	2019	Machine learning techniques	A larger data set is used	BMLT performed worse than expected with mixed genetic and clinical data and some missing data
Tawfik Khoury	2019	Noninvasive scoring	Predicting the presence of intra-abdominal abscesses in patients with emergency CD	Can be used as an adjunct to emergency stratification for patients with acute CD
Jianfeng Pan	2021	Biochemical testing	Identification of patients at risk of thrombosis by D-dimer levels	A predictive model for PTS was constructed to improve the long-term prognosis of CD patients

## 4. Discussion

In the careful sorting of the above existing models, we found that many predictive models for IBDs itself and its extraintestinal symptoms have been proposed and preliminarily verified. However, most of the current prediction models are only useful for validation and applied to small-scale retrospective case analysis, and they are not directly applied to the clinic and lack of self-correction and training of the model. At the same time, we should note that previous IBDs models focused on patients in Europe and the United States, while people from Asia and other developing regions with a high incidence of IBDs were not included in the model. Due to differences in geographical environment, climate, dietary habits, etc., a model of emerging IBDs in high-incidence countries based on underdeveloped regions or even a global model should be rebuilt. We should also be concerned that there is increasing evidence that exercise intensity, fiber intake, and drug use (such as long-term use of contraceptives), previous appendicitis surgery, and psychological factors, which are not directly related to the gastrointestinal tract, also affect the occurrence and development of IBDs. Particular attention should be paid to the psychological factors of patients with chronic disease, which mainly based on information form patient-reported outcomes (PROs). Anxiety and depression brought by unexpected changes, epidemics (67), and sexual disorders related or not related to diseases (68, 69) can not only directly change the intestinal flora or hormone levels of patients (13), but also affect the patient’s treatment compliance (67, 70), thereby causing the treatment of the disease to deviate from daily routine, leading to changes in the course of the disease (71). Previous models have paid more attention to the organic lesions, dominant clinical manifestations, and genes that have already been detected in patients, while the basic life conditions and mental-psychological status closely related to each chronic disease patient have not been considered, especially at certain moments, such as sudden changes, epidemics, or unexpected accidents.

**Figure 2:**
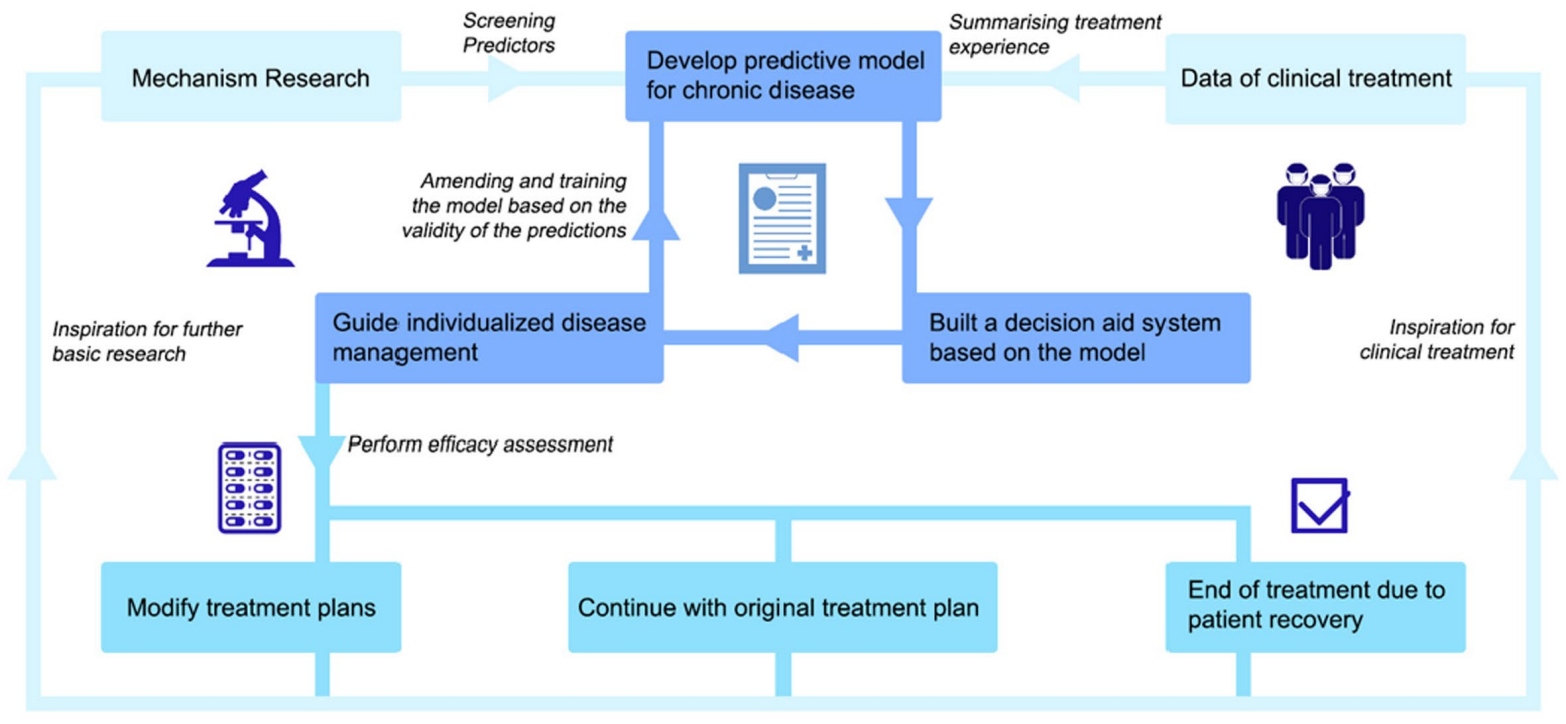
A negative-feedback mechanism based IBDs prediction model. This innovative closed-loop prediction model for patients with IBDs use a “negative feedback” mechanism to continuously modify and train the model. Using “Mechanism Research” and “Data of clinical treatment” as a starting point, we can screen for a certain number of representative predictors. These predictors range from macro to micro and can cover both basic patient information and information at the pathological or genetic level. While relying on the clinical big data platform to evaluate the reliability and value of these predictors, we can build a “predictive model.” Subsequently, we should attempt to “build a decision assistance system.” It could be used to predict disease susceptibility, clinical phenotype, duration of disease, complications, and recurrence risk and severity. In this way, the instructing physician can formulate a targeted diagnosis and treatment plan for the patient according to the predicted result and “apply individualized disease management.” Finally, an efficacy assessment will be performed to determine whether to change the patient’s treatment plan. There are three parallel possible scenarios— “Modify treatment plans”, “Continue with original treatment plan,” and “End of treatment due topatient recovery.” Additionally, the model should be revised and trained according to the validity of the prediction to further improve the decision-making assistance system. These results can also be fed back to basic and clinical research, providing inspiration for further development.

Therefore, we propose a closed-loop prediction model that uses the “negative feedback” mechanism to continuously revise and train the model (Figure 2). We call on researchers to pay attention to the combination of model and clinical data, pay attention to the basic condition, mental-psychological status, and clinical performance of patients, and strive to achieve truly personalized medicine through continuous correction of the model. The information not only comes from medical information systems, but equally critical sources are PROs. The pattern is expressed as follows. On the basis of thorough research on the pathogenesis, epidemiology, and PROs, we can screen for a certain number of representative predictors of IBDs onset, extraintestinal manifestations, and recurrence. These predictors range from macro to micro and can cover both basic patient information and information at the pathological or genetic level. While relying on the clinical big data platform to evaluate the reliability and value of these predictors, we can build a predictive model suitable for IBDs management by relying on electronic health records, basic research databases, and large clinical databases of IBDs patients. If possible, conduct a follow-up visit to the patients in the databases to obtain their PROs, inquiring about some key issues that are not available in clinical information systems. Subsequently, we should attempt to build a decision assistance system. It could be used to predict disease susceptibility, clinical phenotype, duration of disease, complications, and recurrence risk and severity. When a patient sees a doctor, based on certain predictors present in the patient, the decision assistance system can quickly classify the patient into a subgroup of inflammatory bowel diseases and make a prediction by comparing the patient with global patient data. The predicted outcome will include the patient’s likely clinical phenotype, duration of disease, type of complications, and risk and severity of recurrence. In this way, the instructing physician can formulate a targeted diagnosis and treatment plan for the patient according to the predicted result and apply individualized disease management. Finally, an efficacy assessment will be performed to determine whether to change the patient’s treatment plan. Additionally, the model should be revised and trained according to the validity of the prediction to further improve the decision-making assistance system. These results can also be fed back to basic and clinical research, providing inspiration for further development.

From previous studies, we can easily conclude that the establishment of a predictive model for IBDs management is feasible and desirable, in terms of both information sources and methodology. Relying on effective predictors lays a solid foundation for researchers to develop predictive models. In terms of information sources, many factors that can be used to predict the incidence, prognosis, and complications of IBDs and how to obtain them have been determined. With the development of more advanced macroscopic and microscopic research techniques, we believe that there will be more in the future. Predictors closely related to the IBDs have been discovered and confirmed. Integrating different types of scattered predictors to build a more complete and convenient universal model is a breakthrough. In terms of methodology, some progress has been made in research on the prediction model of IBDs itself and other extraintestinal symptoms. Combining its advantages and disadvantages, it can provide a reference for the construction of a systematic IBDs prediction model. Therefore, we believe that it is entirely feasible to build a predictive model related to IBDs and to create a decision-making assistance system on a trial basis. From the disease as a starting point, we can apply this idea to the management of more noncommunicable chronic diseases and use the model of individualized medicine to help patients to better manage chronic diseases.

The original intention of our prediction model was to establish a convenient and individualized medical method to guide clinical diagnosis and treatment. Therefore, we innovatively propose a closed-loop prediction model for IBDs patients who use the “negative feedback” mechanism to continuously revise and train the model. Not limited to IBDs but for all non-infectious chronic diseases. Chronic diseases have a long diagnosis and treatment time, many accumulated organs, frequent complications, easy recurrence, and lack of specificity. These facts require clinicians to strengthen their comprehensive understanding of them in the practice process and to attempt to achieve early detection, early diagnosis, and early treatment. After developing an effective predictive model based on reliable predictors, we not only can predict the patient’s disease outcome and development based on the predictive model but also can achieve early diagnosis and early treatment. It is also possible to conduct individualized disease management and interventions according to the predicted results and use reliable treatment methods, such as drugs and surgery, to intervene in the disease to evaluate the effectiveness of the treatment plan according to whether the intervention results are effective and to what extent or to terminate or revise the treatment plan over time. Negative feedback can act on the original prediction model to realize the revision and training of the model. Therefore, we believe that this closed-loop prediction model will guide the individualized medical treatment of patients, which can realize scientific long-term medication, precise disease management, and individualized medical treatment, effectively prolonging the remission period and reducing morbidity and disability rates. We expect that the coexistence of chronic disease patients and diseases can be realized with this model, thereby achieving a new height of chronic disease management and providing substantial value for its rapid diagnosis and adequate treatment.

Most importantly, we should also note that the study of disease is never the ultimate goal of medicine; our focus is always on preventing disease, reducing morbidity, and promoting and maintaining health. Therefore, we once again call on researchers to pay attention to the combination of models and clinical practice and to the continuous correction of the models, as well as to strive to achieve truly personalized medicine.

## Author contributions

XG, LC, YuC, ZL, JZ, DL, ZJ, YaC, MF, and GY contributed to the conception of the review. XG, LC, and YuC conceived the study subject and made substantive revisions to the important content of the manuscript, and they were the major contributors to the writing of the manuscript. ZL, DL, ZJ, and JZ drew the illustrations, polished the language, and created the tables. YaC provided suggestions and technical support, revised important sections of the manuscript, and assisted in the literature search. GY, ZX, and MF critically reviewed the manuscript. All authors contributed to the article and approved the submitted version.

## Conflict of interest

The authors declare that the research was conducted in the absence of any commercial or financial relationships that could be construed as a potential conflict of interest.

## Publisher’s note

All claims expressed in this article are solely those of the authors and do not necessarily represent those of their affiliated organizations, or those of the publisher, the editors and the reviewers. Any product that may be evaluated in this article, or claim that may be made by its manufacturer, is not guaranteed or endorsed by the publisher.

## Figures and Tables

**Table:** 

IBDs	Inflammatory bowel diseases
CD	Crohn’s disease
UC	Ulcerative colitis
EIMs	Extraintestinal manifestations
CPMs	Clinical prediction models
RF	Random forest
ANNs	Artificial neural networks
GWASs	Genome-wide association studies
CDEIS	Crohn’s disease endoscopic index of severity
SES-CD	Simple endoscopic score for Crohn’s disease
MaRIA	Magnetic resonance index of activity
ROC	Receiver operating characteristic
CDAI	Crohn’s disease activity index
KCDP	Korean Crohn’s disease prediction
NGAL	Neutrophil gelatinase-associated lipid transport protein
MES	Mayo endoscopy score
PPR	Projection pursuit regression
MLTs	Machine learning techniques
ED	Emergency department
PTS	prothrombotic state
CRC	Colorectal cancer
UC-Ca	UC-associated cancer
